# Types of health care facilities and the quality of primary care: a study of characteristics and experiences of Chinese patients in Guangdong Province, China

**DOI:** 10.1186/s12913-016-1604-2

**Published:** 2016-08-02

**Authors:** Ruwei Hu, Yu Liao, Zhicheng Du, Yuantao Hao, Hailun Liang, Leiyu Shi

**Affiliations:** 1 School of Public Health of Sun Yat-sen University, 74 Zhongshan Road II, Guangzhou, China; 2 Johns Hopkins Primary Care Policy Center, Baltimore, 624 N. Broadway, Baltimore, Maryland 21205 USA; 3 Johns Hopkins Bloomberg School of Public Health, 624 N. Broadway, Baltimore, Maryland 21205 USA

**Keywords:** Health policy, Health services development, Health systems, Quality assessment

## Abstract

**Background:**

In China, most people tend to use hospitals rather than health centers for their primary care generally due to the perception that quality of care provided in the hospital setting is superior to that provided at the health centers. No studies have been conducted in China to compare the quality of primary care provided at different health care settings. The purpose of this study is to compare the quality of primary care provided in different types of health care facilities in China.

**Methods:**

A cross-sectional survey with patients was conducted in Guangdong province of China, using the validated Chinese Primary Care Assessment Tool (PCAT). ANOVA was performed to compare the overall and 10 domains of primary care quality for patients in tertiary, secondary, and primary health care settings. Multivariate analyses were used to assess the association between types of facility and quality of primary care attributes while controlling for sociodemographic and health care characteristics.

**Results:**

The final number of respondents was 864 including 161 from county hospitals, 190 from rural community health centers (CHCs), 164 from tertiary hospitals, 80 from secondary hospitals, and 269 from urban CHCs. Type of health care facilities was significantly associated with total PCAT score and domain scores. CHC was associated with higher total PCAT score and scores for first contact-access, ongoing care, comprehensiveness-services available, and community orientation than secondary and/or tertiary hospitals, after controlling for patients’ demographic and health characteristics. Higher PCAT score was associated with greater satisfaction with primary care received. CHC patients were more likely to report satisfactory experiences compared to patients from secondary and tertiary facilities.

**Conclusions:**

The study demonstrated that CHCs provided better quality primary care when compared with secondary and tertiary health care facilities, justifying CHCs as a model of primary care delivery.

**Electronic supplementary material:**

The online version of this article (doi:10.1186/s12913-016-1604-2) contains supplementary material, which is available to authorized users.

## Background

Primary care refers to first-contact, continuous, comprehensive, and coordinated care provided to individuals regardless of gender, disease, or organ system affected [1]. It has been demonstrated that effective primary care is associated with improved access to health care services, reduced hospitalizations, cost effectiveness, and enhanced equity [2–6]. Various factors are associated with primary care quality. Previous research has examined the relationship between types of health care facilities and quality of primary care [7–11]. Comparisons among primary care visits in US health care settings have found that health centers generally achieve higher quality of primary care and that primary care in hospitals is associated with less continuity [7, 8, 11]. However, in China, most people tend to use hospitals rather than health centers for their primary care generally due to the perception that quality of care provided in the hospital setting is superior to that provided at the health centers. This paper assesses the quality of primary care across different Chinese health care facilities and discusses implications of the findings for further enhancing primary care performance in China.

The Chinese health care system is organized as a three-tiered system, which includes tertiary medical centers (including teaching hospitals), secondary hospitals (including urban district hospitals and rural county hospitals), and community health centers (CHCs) (including rural township hospitals) [12]. Despite this classification, patients may seek medical treatment, including primary care, from all three levels without referral [13]. The data from China Ministry of Health (now renamed as Commission of Public Health and Family planning) suggests that visits and admissions continue to take place mainly at secondary and tertiary hospitals in China. According to the 2012 data, more than 36 % of the outpatient services happened in the hospitals [14]. Such a fragmented and uncoordinated health care delivery system leads to unaffordable access and higher cost in China [15, 16]. Health care expenditures are rising faster than income. The annual growth rates in per capita total health care expenditures and gross domestic product were 14.9 and 10.2 %, respectively, between 2007 to 2012 [17].

In 2009, China launched a nationwide systemic health care reform, in which the government explicitly set the goal of strengthening primary care [18]. Under this reform, 2200 county hospitals and more than 330,000 CHCs or rural township hospitals had been reconstructed or upgraded [18]. Providing care in close proximity to the population would reduce barriers of cost and transportation in accessing care. In addition, all provinces adopted the National Essential Medicines List in primary health care facilities by 2011 [18].

However, despite the investment in community-based primary care, many patients still seek primary care from secondary and tertiary settings. Existing literature points out that one of the reasons that patients prefer secondary or tertiary hospitals is that they did not trust the quality provided in CHCs [19]. Do hospitals really provide better quality of primary care than CHCs? We did the literature search in order to obtain related evidence on this. The literature search was done through Pubmed using the following search terms: quality, primary care, health care facility, hospital, community health centers, and China. The search was limited to English language journals and to the period from January 2000 to December 2014. We found that no studies had been conducted in China to compare the quality of primary care provided at different health care settings. The purpose of this study was to fulfill this gap in literature by examining the quality of primary care provided in tertiary, secondary, and primary health care settings in China. Results of the study would provide implications for policymakers in terms of improving primary care performance in China and for consumers in guiding their health care seeking behavior.

## Methods

### Study design and participants

The study was carried out in Guangdong province of southern China, which is a coastal province with a population of more than 100 million. With 30 % of its total population being migrants, Guangdong is the most populous province accounting for the largest number of internal migrant population in China [20]. With a rapid pace in economic development, Guangdong plays a leading role in trend-setting implementation of health policy initiatives including the development of a primary care infrastructure [20]. Thus, Guangdong affords an ideal study site to assess the impact of types of health care facilities on the quality of primary care.

We conducted a cross-sectional survey with patients whose usual source of primary care was the study site. The sample size was calculated based on findings from a previous paper that compared the PCAT scores between patients at a health maintenance organization and patients at a CHC [21]. The minimum sample size of this study was estimated as 800 with a 99 % confidence interval and a power of 80 %.

A multistage cluster sampling method was adopted. In the first stage, all cities in Guangdong province were categorized into two levels: developed or developing city, according to the per capita GDP. In each level, we randomly selected two cities. In each city, we included 200 patients. In the second stage, we stratified between rural and urban areas within each city. The sampled rural areas were adjacent to the selected urban areas. In rural areas, we enrolled patients in county hospitals and rural CHCs, while in urban areas we enrolled patients in tertiary hospitals, secondary hospitals and urban CHCs. The selection of study sites was based on purposive sampling, with input from our local research partner, faculty from the School of Public Health at the Sun Yat-sen University. Thus, there were 50 patients randomly enrolled from each type of health care facility. The study subjects were individuals aged 18 or over who visited either CHCs or hospitals in these four cities. Additional file 1 illustrates the process of multistage cluster sampling. The final number of included respondents was 864. There were 161 respondents from county hospitals, 190 from rural CHCs, 164 from tertiary hospitals, 80 from secondary hospitals, and 269 from urban CHCs.

Researchers from the School of Public Health of Sun Yat-sen University in Guangdong, China, conducted the primary data collection. Informed consent was obtained from all participating study subjects. The Institutional Review Board (IRB) of Sun Yat-sen University reviewed and approved the protocol of the study in compliance with the Declaration of Helsinki – Ethical Principles for Medical Research Involving Human Subjects (Approval No.: IRB2014.9).

### Measures

We used the validated Chinese Primary Care Assessment Tool (PCAT) to measure the extent and quality of primary care services [22]. The PCAT was administered through face-to-face interviews between November 2013 and September 2014. On average, the survey required 20 min to administer. One of the advantages of PCAT is that it does not rely on respondents’ expectations, perceptions, or values. Rather than ratings of perception or satisfaction, PCAT items measure patients’ actual experiences with different aspects of their primary care visits [23]. PCAT is theoretically and practically scientific. A series of validation analyses of PCAT were conducted worldwide. Studies reported high reliability, construct and content validity for this instrument regarding actual patient experiences [24–27]. The Chinese PCAT has also been validated and found highly valid and reliable after some minor rewording of items in the comprehensiveness domain due to contextual differences [22]. The instrument measures ten sub-domains of seven key features of primary care performance. The PCAT items assesses patients’ self-perceived experiences of primary care in ten scales: first contact (two sub-domains) is defined as the accessibility to and use of primary care services when a new health or medical problem arises; continuity of care refers to the longitudinal use of a regular source of primary care over time; coordination (two sub-domains) refers to the interpersonal linkage of care between different levels of providers or informational linkage of care through using the electronic information system; comprehensiveness (two sub-domains) refers to the availability of clinical and preventive services within the provider; family centeredness is defined as the inclusion of family health concerns in decision-making; community orientation refers to the provider’s knowledge of community health needs; and cultural competence is defined as patients’ willingness to recommend their primary care provider to others [24]. A 4-point Likert-type scale was applied to measure certainty as to whether a service was received, coded as “1” ("Definitely Not"), “2” (Probably not), “3” (Probably) “4” (“Definitely”). A neutral response of “Not sure/don’t remember” was provided for the lack of knowledge about a characteristic. The PCAT items yielded a primary care performance total score and ten primary care performance scale scores. The total score was the sum of ten primary care domain scores. The measure of patient satisfaction was dichotomous, defined as whether the patient was satisfied with their current health care provider.

### Analysis

The overall aim of the analysis was to compare the quality of primary care experienced by patients in different types of health care facilities. First, chi-square analyses were applied to compare socio-demographic characteristics and health status of patients in different types of health care facilities. Next, ANOVA was performed to compare quality of care indicators for patients in different types of health care facilities. Multiple linear regression models were then used to assess the association between types of facility and quality of primary care attributes after controlling for patients’ socio-demographic, health, and health care characteristics. The covariates were extracted based on the Aday and Andersen’s access-to-care framework [28]. Finally, logistic regression models were used to assess the association between types of facilities, quality of primary care, and patient satisfaction, controlling for patients’ socio-demographic, health, and health care characteristics.

## Results

### Demographic characteristics of primary care patients

Table 1 provides the demographic characteristics of the subjects included in this study, and compares the frequency and percentage distribution of primary care visits according to patient characteristics for the five types of health care facilities. Overall, a greater proportion of primary care visits were from females than from males (59.1 % vs 40.9 %). The majority of visits were from patients aged 18 to 64 years (72.6 %). Both rural CHCs and urban CHCs had a higher proportion of visits from permanent resident individuals (65.8 and 57.6 %, respectively). Compared with the other types of health care facilities, tertiary hospital users had larger proportions of patients with higher education, employment and income level. In contrast, many patients in urban CHCs were unemployed (65.1 %). In rural CHCs, the sample included more patients with education level less than middle school (73.4 %) and with lower income level (42.6 %). However, both rural and urban CHCs had a lower proportion of visits from uninsured patients (33.2 and 27.5 %, respectively), compared to county (52.2 %), secondary (45 %), and tertiary hospitals (40.9 %).

**Table 1:** Demographic, socioeconomic, and health measures of the respondents in Guangdong Province by type of healthcare facilities

	Total	County hospital	Rural CHC	Tertiary hospital	2ndary hospital	Urban CHC
	N (%)	N (%)	N (%)	N (%)	N (%)	N (%)
N	796 ~ 864	136 ~ 161	179 ~ 190	153 ~ 164	73 ~ 80	255 ~ 269
Sex**						
Female	511 (59.1)	83 (51.6)	103 (54.2)	90 (54.9)	54 (67.5)	181 (67.3)
Male	353 (40.9)	78 (48.4)	87 (45.8)	74 (45.1)	26 (32.5)	88 (32.7)
Age**						
< 18	126 (14.6)	37 (23.0)	37 (19.5)	20 (12.2)	7 (8.8)	25 (9.3)
18 ~ 44	125 (14.5)	13 (8.1)	20 (10.5)	12 (7.3)	11 (13.8)	69 (25.7)
45 ~ 64	376 (43.5)	87 (54.0)	65 (34.2)	99 (60.4)	36 (45.0)	89 (33.1)
≥ 65	237 (27.4)	24 (14.9)	68 (35.8)	33 (20.1)	26 (32.5)	86 (32.0)
Marital status**						
Not married	261 (30.2)	65 (40.4)	65 (34.2)	53 (32.3)	19 (23.8)	59 (21.9)
Married	603 (69.8)	96 (59.6)	125 (65.8)	111 (67.7)	61 (76.2)	210 (78.1)
Residence**						
Rural	351 (40.6)	161 (100)	190 (100)	0 (0)	0 (0)	0 (0)
Urban	513 (59.4)	0 (0)	0 (0)	164 (100)	80 (100)	269 (100)
Migrant**						
Yes	400 (46.3)	89 (55.3)	65 (34.2)	90 (54.9)	42 (52.5)	114 (42.4)
No	464 (53.7)	72 (44.7)	125 (65.8)	74 (45.1)	38 (47.5)	155 (57.6)
Education**						
≤ Middle school	402 (47.3)	73 (45.9)	135 (73.4)	40 (24.7)	37 (47.4)	117 (44.0)
High school	168 (19.8)	29 (18.2)	32 (17.4)	24 (14.8)	14 (17.9)	69 (25.9)
Technical/college	182 (21.4)	32 (20.1)	16 (8.7)	54 (33.3)	21 (26.9)	59 (22.2)
≥ Undergraduate	97 (11.4)	25 (15.7)	1 (0.5)	44 (27.2)	8 (7.7)	21 (7.9)
Occupation**						
Unemployed	438 (50.7)	85 (52.8)	81 (42.6)	55 (33.5)	42 (52.5)	175 (65.1)
Farmer	101 (11.7)	15 (9.3)	49 (25.8)	9 (11.2)	9 (11.2)	19 (7.1)
Worker	325 (37.6)	61 (37.9)	60 (31.6)	29 (36.2)	29 (36.2)	75 (27.9)
Insurance**						
Uninsured	324 (37.5)	84 (52.2)	63 (33.2)	67 (40.9)	36 (45.0)	74 (27.5)
Rural	195 (22.6)	69 (42.9)	126 (66.3)	0 (0)	0 (0)	0 (0)
Urban	297 (34.4)	0 (0)	0 (0)	77 (47.0)	38 (47.5)	182 (67.7)
Other	48 (5.6)	8 (5.0)	1 (0.5)	20 (12.2)	6 (7.5)	13 (4.8)
Income**						
Low	160 (18.5)	33 (20.5)	81 (42.6)	9 (5.5)	10 (12.5)	27 (10.0)
Median	475 (55.0)	82 (50.9)	85 (44.7)	89 (54.3)	42 (52.5)	177 (65.8)
High	161 (18.6)	21 (13.0)	13 (6.8)	55 (33.5)	21 (26.2)	51 (19.0)
Health status**						
Fair/poor	347 (40.2)	43 (26.7)	88 (46.3)	64 (39.0)	29 (36.2)	123 (45.7)
G/VG/E	517 (59.8)	118 (73.3)	102 (53.7)	100 (61.0)	51 (63.8)	146 (54.3)
Chronic condition**						
No	661 (76.5)	138 (85.7)	147 (77.4)	130 (79.3)	60 (75.0)	186 (69.1)
Yes	203 (23.5)	23 (14.3)	43 (22.6)	34 (20.7)	20 (25.0)	83 (30.9)

*P < .05. **p < .01, based on Chi-square test of difference across healthcare settings

### Primary care attributes

Table 2 presents the bivariate results between type of health care settings and the primary care attribute scores (PCAT). Respondents in rural CHCs reported significantly higher total PCAT score (29.50) when compared with those in county (26.95), secondary (27.83) and tertiary hospitals (27.75). In terms of the mean value for total PCAT score and total mean scores in each domain, the cultural competence domain received the highest score (3.22), while the community orientation domain received the lowest score (2.06). The other values for mean scores in each domain were summed up as follows: 3.05 for first contact-utilization, 3.01 for family-centeredness, 3.00 for coordination-information systems, 2.99 for comprehensiveness-services available, 2.86 for comprehensiveness-service used, 2.77 for coordination, 2.75 for first contact-access, and 2.67 for ongoing care. Comparing scores among five types of health care facilities, respondents in rural CHCs reported significantly higher scores for first contact-access, coordination, comprehensiveness services available, family-centeredness, and community orientation domains. Respondents in urban CHCs reported significantly higher scores for ongoing care, coordination of information systems, and cultural competence domains. Comparing scores reported by the respondents from rural health care facilities, respondents from rural CHCs ranked significantly higher for four out of ten domains as well as the total PCAT score than respondents from rural hospitals, including first contact-access (3.03 vs. 2.55, *p* < 0.05), ongoing care (2.73 vs. 2.34, *p* < 0.05), coordination (2.93 vs. 2.68, *p* < 0.05), comprehensiveness-services available (3.13 vs. 2.85, *p* < 0.05) and the total PCAT score (29.50 vs. 26.95, *p* < 0.05). Similar results were observed in urban health care facilities. Respondents from urban CHCs ranked significantly higher for ongoing care, comprehensiveness-services available, and community orientation.

**Table 2:** Individual and total primary care attributes scores reported by respondents by type of healthcare settings

	Total	County hospital	Rural CHC	Tertiary hospital	Secondary hospital	Urban CHC
	Mean (SE)	Mean (SE)	Mean (SE)	Mean (SE)	Mean (SE)	Mean (SE)
Sample size	864	161	190	164	80	269
First Contact-utilization	3.05 (0.64)	3.00 (0.53)	3.13 (0.63)	2.99 (0.67)	3.04 (0.66)	3.06 (0.68)
First Contact-access	2.75 (0.71)	2.55 (0.75)*^#^	3.03 (0.69)*^^@^	2.68 (0.72)^	2.90 (0.68)^#^	2.69 (0.64)^@^
Ongoing Care	2.67 (0.75)	2.34 (0.76)*^#^^^@^	2.73 (0.66)*	2.60 (0.73)^#@^	2.70 (0.80)^	2.86 (0.74)^@^
Coordination	2.77 (0.68)	2.68 (0.57)*	2.93 (0.56)*^#^	2.68 (0.82)^#^	2.81 (0.68)	2.77 (0.72)
Coordination-information systems	3.00 (0.67)	2.83 (0.78)*^#^	2.89 (0.72)^	3.08 (0.55)*	2.95 (0.69)^#^	3.13 (0.60)^
Comprehensiveness-serv. available	2.99 (0.56)	2.85 (0.56)*^#^	3.13 (0.51)*^	2.97 (0.56)	2.81 (0.48)^^@^	3.03 (0.60)^#@^
Comprehensiveness-service used	2.86 (0.76)	2.80 (0.64)	2.97 (0.79)	2.77 (0.78)	2.81 (0.78)	2.88 (0.77)
Family-centeredness	3.01 (0.90)	3.01 (0.85)	3.17 (0.76)*	3.01 (0.97)	2.94 (0.97)	2.91 (0.95)*
Community Orientation	2.06 (0.82)	1.78 (0.71)*	2.32 (0.84)^#^^	1.77 (0.74)^#@^	1.76 (0.75)^^$^	2.31 (0.79)*^@$^
Cultural Competence	3.22 (0.65)	3.12 (0.80)*	3.20 (0.64)	3.20 (0.58)	3.11 (0.55)	3.31 (0.62)*
Total PCAT score	28.37 (4.52)	26.95 (3.94)*^#^	29.50 (4.53)*^^@^	27.75 (4.28)^	27.83 (4.71)^@^	28.96 (4.64)^#^

Significance indicated at *p* < .05, based on Bonferroni post-hoc means test

^*, #, ^, @, $^indicate the significant differences. Results with similar symbols indicate significant differences between settings

The relationships between types of health care setting and the primary care scores is displayed on the radar chart. The chart visualizes the PCAT scores in ten sub-domains reported by patients from three different types of health care facilities on a scale from 1 to 4. It also gives an overall sense of each group’s ratings. From this chart, it is apparent that patients in CHCs reported higher PCAT score than those in secondary and tertiary hospitals. The chart also provides the details for each domain. The quality of primary care in different types of health care facilities was predominantly different, with statistically significant values for the domain of first contact-access, ongoing care, coordination-referrals, coordination-information systems, comprehensiveness-services available, community orientation and culturally competency (*p* < 0.01), and comprehensiveness-services used (*p* < 0.05).

### Multivariate analyses of primary care attributes

Table 3 presents the multivariable linear regression results between the patient/institutional characteristics and primary care attributes. Multivariate analyses were used to assess the association between type of primary care facilities and quality of primary care after controlling for patients’ demographic and health characteristics. The type of health care facilities was significantly associated with total PCAT score. With CHCs as the reference group, the coefficient for secondary hospitals was −1.50, and −0.95 for tertiary hospitals. Thus, respondents in CHCs would have on average an estimated 1.5 points greater score than those in secondary hospitals, and 0.95 greater score than those in tertiary hospitals. Similarly, the results also showed the type of health care facilities was also predictive of scores in ongoing care and community orientation domains. In the ongoing care domain, the score of CHCs was 0.21 higher than secondary and tertiary hospitals, respectively (*p* < 0.01). In the community orientation domain, the score of CHCs was 0.54 and 0.51 higher than secondary and tertiary hospitals, respectively (*p* < 0.01).

**Table 3:** Patient and institutional characteristics associated with individual and total primary care attributes (*N* = 864)

	First contact-utilization	First contact-access	Ongoing care	Coordination	Coordination-information systems	
	B (SE)	B (SE)	B (SE)	B (SE)	B (SE)	
CONSTANT	3.64** (0.23)	2.87** (0.25)	2.53** (0.27)	2.72** (0.25)	2.85** (0.24)	
SETTING (Ref: CHC)						
Secondary hospital	−0.01 (0.05)	−0.11* (0.06)	−0.21** (0.06)	−0.08 (0.06)	−0.03 (0.05)	
Tertiary hospital	0001 (0.06)	−0.10 (0.07)	−0.21** (0.07)	−0.11 (0.07)	0.06 (0.07)	
Sex (Ref: Female)						
Male	−0.14** (0.04)	−0.14** (0.05)	−0.09 (0.05)	−0.03 (0.05)	−0.12** (0.05)	
Age (Ref: ≥65)						
45 ~ 64	−0.2** (0.07)	0.02 (0.08)	−0.05 (0.08)	−0.05 (0.08)	−0.17* (0.07)	
18 ~ 44	−0.21** (0.08)	0.08 (0.09)	−0.14 (0.09)	−0.01 (0.09)	−0.27** (0.08)	
≤ 18	−0.12 (0.10)	0.14 (0.11)	−0.25* (0.11)	−0.05 (0.11)	−0.24* (0.10)	
Marital status (Ref.: single)						
Married	0.01 (0.06)	0.04 (0.07)	−0.14* (0.07)	−0.10 (0.07)	−0.11 (0.06)	
Location (Ref.: Rural)						
Urban	0.11* (0.07)	0.20* (0.08)	0.26** (0.08)	0.14 (0.08)	0.34** (0.08)	
Migrant (Ref.: Yes)						
No	−0.03 (0.05)	−0.05 (0.05)	0.01 (0.05)	−0.06 (0.05)	−0.08 (0.05)	
Education (Ref.: Middle school)						
High school	−0.12 (0.06)	0.02 (0.07)	−0.07 (0.07)	0.04 (0.06)	0.03 (0.06)	
Technical/college	0.08 (0.06)	0.20** (0.07)	0.10 (0.07)	0.17* (0.07)	0.14* (0.06)	
≥ Undergraduate	0.05 (0.08)	0.16 (0.09)	0.04 (0.09)	0.04 (0.09)	0004 (0.08)	
Occupation (Ref.: Unemployed)						
Farmer	0.05 (0.08)	0.15 (0.08)	−0.04 (0.09)	0.10 (0.08)	−0.09 (0.08)	
Worker	−0.06 (0.06)	−0.05 (0.06)	−0.11 (0.07)	−0.04 (0.06)	−0.09 (0.06)	
Insurance (Ref.: Uninsured)						
Rural	0.27** (0.07)	0.59** (0.08)	0.32** (0.08)	0.42** (0.08)	0.51** (0.07)	
Urban	0.10 (0.06)	0.12 (0.07)	0.13 (0.07)	0.12 (0.07)	0.15* (0.06)	
Other	−0.21** (0.10)	0.14 (0.11)	0.07 (0.12)	−0.02 (0.11)	0.06 (0.10)	
Income (Ref.: Low)						
Median	−0.13* (0.06)	−0.13 (0.07)	0.01 (0.07)	−0.08 (0.07)	0001 (0.07)	
High	−0.30** (0.08)	−0.29** (0.09)	−0.04 (0.09)	−0.21* (0.09)	−0.04 (0.08)	
Health status (Ref.: Fair/poor)						
G/VG/E	0.02 (0.05)	−0.06 (0.05)	0.09 (0.05)	0.13* (0.05)	0.11* (0.05)	
Chronic conditions (Ref.: No)						
Yes	−0.25** (0.06)	−0.19** (0.06)	0.05 (0.06)	−0.06 (0.06)	−0.03 (0.06)	
Adjust R square	0.091	0.120	0.120	0.069	0.122	
N	864	864	864	864	864	
	Comprehensiveness-services available	Comprehensiveness-service used	Family-centeredness	Community orientation	Cultural competence	Total PCAT score
	B (SE)	B (SE)	B (SE)	B (SE)	B (SE)	B (SE)
CONSTANT	2.98** (0.21)	3.02** (0.28)	3.27** (0.33)	2.35** (0.29)	2.68 (0.24)	28.92** (1.58)
SETTING (Ref.: CHC)						
Secondary hospital	−0.24** (0.05)	−0.09 (0.06)	−0.04 (0.08)	−0.54** (0.07)	−0.14 (0.05)	−1.50** (0.36)
Tertiary hospital	−0.06 (0.06)	−0.08 (0.08)	0.06 (0.09)	−0.51** (0.08)	0.004 (0.07)	−0.95* (0.44)
Sex (Ref.: Female)						
Male	−0.08 (0.04)	−0.08 (0.05)	−0.12 (0.06)	−0.04 (0.05)	−0.11 (0.05)	−0.94** (0.30)
Age (Ref.: ≥65)						
45 ~ 64	0.02 (0.06)	−0.04 (0.09)	−0.09 (0.10)	−0.06 (0.09)	0.03 (0.07)	−0.60 (0.49)
18 ~ 44	0.02 (0.07)	−0.05 (0.10)	−0.05 (0.12)	−0.01 (0.10)	−0.04 (0.08)	−0.69 (0.55)
≤ 18	0.10 (0.09)	−0.15 (0.12)	0.21 (0.14)	−0.18 (0.12)	−0.06 (0.10)	−0.60 (0.67)
Marriage (Ref.: Single)						
Married	0.01 (0.05)	−0.05 (0.07)	−0.01 (0.09)	−0.16* (0.07)	0.10 (0.06)	−0.41 (0.41)
Location (Ref.: Rural)						
Urban	−0.08* (0.07)	0.02 (0.09)	−0.08 (0.11)	−0.05 (0.09)	−0.10 (0.07)	0.76 (0.50)
Migrant (Ref.: Yes)						
No	−0.05* (0.04)	−0.07 (0.06)	−0.1 (0.07)	−0.12* (0.06)	0.08 (0.05)	−0.47 (0.31)
Education (Ref.: ≤Middle school)						
High school	0.04 (0.05)	0.01 (0.07)	−0.04 (0.09)	0.004 (0.07)	−0.01 (0.06)	−0.11 (0.41)
Technical/college	0.10 (0.05)	0.13 (0.07)	0.17 (0.09)	0.20** (0.07)	0.07 (0.06)	1.36** (0.41)
≥ Undergraduate	0.07 (0.07)	0.03 (0.10)	0.09 (0.12)	−0.02 (0.10)	−0.02 (0.08)	0.44 (0.55)
Occupation (Ref.: Unemployed)						
Farmer	0.06 (0.07)	0.15 (0.09)	0.30** (0.11)	0.13 (0.10)	−0.15 (0.08)	0.67 (0.53)
Worker	0.02 (0.05)	0.01 (0.07)	0.09 (0.08)	−0.04 (0.07)	−0.14 (0.06)	−0.40 (0.40)
Insurance (Ref.: Uninsured)						
Rural	0.26 (0.06)	0.32** (0.09)	0.32** (0.1)	0.18* (0.09)	−0.01 (0.07)	3.17** (0.49)
Urban	0.21** (0.06)	0.20** (0.08)	0.22* (0.09)	0.22** (0.08)	0.09 (0.06)	1.56** (0.42)
Other	0.20** (0.09)	0.06 (0.12)	0.08 (0.14)	0.24* (0.12)	−0.11 (0.10)	0.52 (0.68)
Income (Ref.: Low)						
Median	−0004 (0.06)	−0.04 (0.08)	−0.06 (0.09)	0.03 (0.08)	0.16 (0.07)	−0.24 (0.44)
High	−0.02 (0.07)	−0.17 (0.10)	−0.20 (0.12)	−0.23* (0.10)	0.19 (0.08)	−1.31* (0.55)
Health status (Ref.: Fair/poor)						
G/VG/E	0.06 (0.04)	0.13* (0.06)	0.14* (0.07)	0.23** (0.06)	0.24 (0.05)	1.09** (0.32)
Chronic condition (Ref.: No)						
Yes	0.06 (0.05)	−0.11 (0.07)	−0.14 (0.08)	0.07 (0.07)	0.11 (0.06)	−0.49 (0.38)
Adjust R square	0.061	0.043	0.078	0.153	0.065	0.147
N	864	864	864	864	864	864

*p < .05, **p < .01

We also fit multivariable logistic regression models to examine factors associated with patients’ satisfaction (Table 4). Significant association between PCAT total score and patients’ satisfaction was observed. The results indicated that respondents were more likely to be satisfied with primary care if they reported higher PCAT score. The probability of patients being satisfied with primary care increased by 1.22 times (*p* < 0.01) as the PCAT score increased by 1 unit. A significant association between type of health care facilities and patients’ satisfaction with care was also observed. The results showed that patients in CHCs were more likely to report satisfaction with care than patients in secondary (0.60, 95 % CI = 0.38–0.94) and tertiary hospitals (0.54, 95 % CI = 0.30–0.98). In addition, other covariates were also significantly associated with satisfaction of primary care, including age, location, household registration and income. Specifically, younger patients, residents of urban areas, migrants, and higher income patients reported greater satisfaction with primary care than their counterparts.

**Table 4:** Factors associated with patients’ satisfaction with care (*N* = 864)

Measures	OR (95 % CI)
PCAT total score	1.22** (1.16,1.28)
SETTING (Ref: CHC)	
Secondary hospital	0.60* (0.38,0.94)
Tertiary hospital	0.54* (0.30,0.98)
Sex (Ref.: Female)	
Male	1.37 (0.92,2.05)
Age (Ref.: ≥65)	
45 ~ 64	0.88 (0.40,1.93)
18 ~ 44	0.59 (0.26,1.31)
≤ 18	0.30** (0.2,0.80)
Marital status (Ref.: Single)	
Married	0.73 (0.41,1.30)
Location (Ref. Rural)	
Urban	2.67** (1.44,4.90)
Migrant (Ref.: No)	
Yes	1.88** (1.23,2.88)
Education (≤Middle school)	
High school	0.74 (0.43,1.25)
Technical/college	0.74 (0.43,1.28)
≥ Undergraduate	1.14 (0.57,2.31)
Occupation (Ref.: Unemployed)	
Farmer	1.15 (0.55,2.44)
Worker	0.99 (0.59,1.66)
Insurance (Ref.: Uninsured)	
Rural	0.94 (0.53,1.67)
Urban	0.69 (0.38,1.24)
Other	0.64 (0.27,1.53)
Income (Ref.: Low)	
Median	2.26** (1.30,3.92)
High	1.97 (0.99,3.92)
Health status (Ref.: Fair/Poor)	
G/VG/E	1.27 (0.84,1.92)
Chronic condition (Ref.: No)	
Yes	1.16 (0.69,1.97)
Constance	0.002**
Nagelkerke R square	0.263

*p < .05, **p < .01

## Discussion

This study was one of the first to evaluate and compare the quality of primary care among different types of health care settings in China using an internationally recognized and locally validated tool, PCAT. The study added evidence that CHCs could provide better or equal primary care when compared with other health care providers in China, and supported the appropriateness of the CHC delivery model in providing primary care not only to vulnerable populations but also to the entire population.

Results from this study showed that women and those unemployed were more likely to be CHC patients compared to tertiary hospitals which had a greater proportion of patients from higher education, employment, and income levels. The study showed the potential for CHCs to help bridge the disparities faced by vulnerable populations in terms of longer travel and wait times, limited patient-provider contact, and weak continuity of care.

CHCs received higher PCAT scores than secondary and tertiary hospitals. The largest difference of total PCAT scores between the highest- and lowest-performing facilities were 2.55 (29.50 for rural CHCs vs. 26.95 for county hospitals, *p* < 0.05), which suggested CHCs in rural area generally were perceived to provide higher level quality of care compared to the hospital settings. Rural CHCs received higher scores for first contact access, coordination, comprehensive services, family centeredness, and community orientation. The largest difference of PCAT domain scores between the highest- and lowest-performing facilities in rural area was 0.48 in first contact-assess, which suggested that it was easier and more convenient for patients to access CHCs in rural area, and which may be explained by the following factors: convenient travel distance to CHCs, no appointments required, and shorter waiting time. Urban CHCs rated better in continuity of care, coordination with information systems, and cultural competency. CHC patients were more likely to report satisfactory experiences compared to secondary and tertiary patients.

In part, these findings may be explained by the different finance and incentive methods between different types of health care facilities. CHCs in China are now mostly financed by government subsidies to provide primary care, thus the service are provided with stronger and better policy implementation [29], such as the establishment of chronic disease management and family practice service program, which would likely improve patient’s satisfaction with their primary care experience [30, 31]. The other explanation could be due to China’s current health care reform policies to strengthen its primary care infrastructure. The reform invested in infrastructure and provider training, and established the Essential Medicine Program in CHCs to improve the safety, quality and efficiency of primary care services [18]. Besides, it should be noted that care continuity is particularly important for primary care patients, as primary care patients are more likely to use health care frequently and can benefit from a closer patient-physician relationship. Our study showed CHCs ranked better in continuity of care. As previous studies showed that smaller practices had higher continuity [32], our findings may be explained by that CHCs are smaller than hospitals and this could lead to better relational continuity between the physician and the patient and help providers continuously contact patients for follow-up care.

**Fig. 1:**
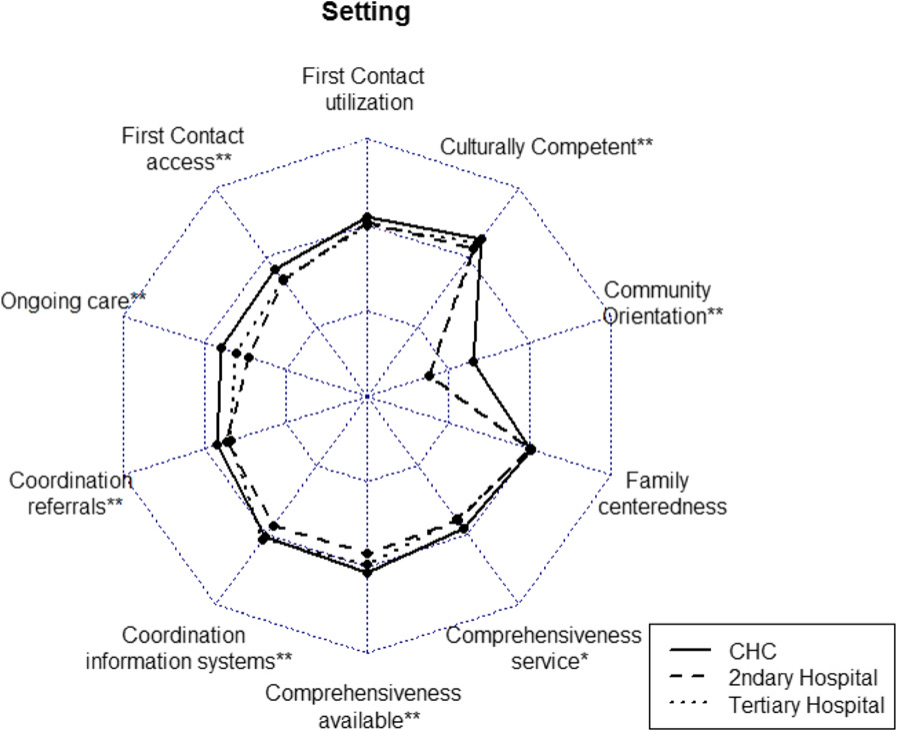
Radar chart plots: primary care attributes scores reported by respondents by type of healthcare settings

Our results affirmed the CHC model as an appropriate one for the delivering of primary care, which are also consistent with previous studies in other countries. The studies in the United States have credited the community health center model with providing accessible, cost-effective, and high quality primary care and reducing health disparities [33–36]. The absolute differences in domain and total PCAT scores across different facility types are small, which is comparable to previous study conducted in other regions of China [37]. Besides, the pattern of relative scores across domains was also comparable to other Chinese studies using PCAT [38]. A study conducted in Shenzhen and Shanghai reported that higher score in coordination-information and comprehensiveness-services available across eight primary care domains [38]. These two domain scores also ranked as two of the top five across ten domains in our study. From Fig. 1 it was clear that patients from all three types of health care facilities reported lower scores for the community orientation domain. One possible explanation is that China’s current health care system is a hospital-centric, rather than a primary health care-centered integrated delivery model; which leads to the challenge to transform primary health care delivery from an approach based on patients and episodes to a population- and community-based approach.

There were a number of limitations in this study. First, we used cross-sectional data, which would be an efficient way to obtain a large sample. However, it would be difficult to make causal inferences from the analysis. Second, the survey data were based entirely on self-reports and thus subject to recall bias. Third, there could be unmeasured confounders leading to potential residual confounding of data. Fourth, this study examined the patient perceived experiences rather than the health status outcomes of primary health care services, such as disease-specific technical aspects of provider practice. And the variation of patient perceived experiences may be attributed to differences in patients’ characteristics. Further research is needed to investigate if types of health care facilities are related to better health outcomes, and to include clinical quality measures derived from electronic medical record abstraction or clinical vignettes.

## Conclusions

Despite these limitations, the findings of this study are helpful in informing policy decisions. The study affirmed CHCs as a model to provide better or equal quality primary care when compared with other health care facilities, which would encourage patients to seek primary care in CHCs. CHCs play a critical role in China’s primary care delivery system. An adequately funded and well-organized health center system can play a gatekeeping role and has the potential to provide a reasonable level of care to patients, especially vulnerable sub-populations. In China’s current nationwide systemic health care reform, primary care should play a central role in the face of increasing pressure from demographic, epidemiological and socioeconomic forces. Our study affirmed a positive statistical association between seeking care in CHCs’ and primary care attributes in continuity, community orientation, comprehensiveness-services available, and first contact-access. The policy implication is to continue the ongoing efforts in strengthening primary health care. One of the health care reform priorities is to rebuild an integrated health care delivery system centered around primary care, focusing on population health and community benefits rather than on individual patient or episode. Effective strategies include moving toward pay-for-performance and away from pay-per-service, centering primary care facilities as the main coordinators of care, establishing formal referral arrangements, information sharing mechanisms, and proper incentives for both patients (eg, lower charges and speedier access) and providers (eg, administratively and performance linked) to encourage greater utilization of CHC for primary care. Additionally, the public’s perception of low quality care in community-based primary care should be altered. The policy implication is to address concerns of patients by improving medical education for primary care oriented providers and enhancing the skill sets for current workforce at the CHCs level, and improving the skill areas of providers (through training, educational programs, etc.). Multimedia campaigns can also serve as a tool to disseminate evidence of quality care and greater patient satisfaction in CHCs. Word of mouth is a widely trusted source of information. Encouraging patients to refer friends and family to CHCs is another method to demystify the quality of community primary care. Actively improving patient care-seeking behaviors and the delivery of primary care contributes to maintaining and improving the health of populations through a coordinated, comprehensive, and continuous health care model.

## Abbreviations

CHCs, Community health centers; IRB, Institutional Review Board; PCAT, Primary Care Assessment Tool

## Additional file

Additional file 1: Multistage Cluster Sampling Method. (DOCX 125 kb)
