# Supplementary material for: Types of health care facilities and the quality of primary care: a study of characteristics and experiences of Chinese patients in Guangdong Province, China
Source: BMC Health Serv Res. 2016 Aug 2;16:335. doi: 10.1186/s12913-016-1604-2 (PMC4969734; doi:10.1186/s12913-016-1604-2)
Supplement: Additional file 1: — Multistage Cluster Sampling Method. (DOCX 125 kb) [file 12913_2016_1604_MOESM1_ESM.docx]

Appendix 1. Multistage Cluster Sampling Method

Urban CHCs

Tertiary hospitals

Rural CHCs

Tertiary hospitals

Urban CHCs

Tertiary hospitals

Urban CHCs

Rural CHCs

Rural CHCs

Tertiary hospitals

Urban CHCs

Rural CHCs

Secondary hospitals

Secondary hospitals

Secondary hospitals

Secondary hospitals

County hospitals

County hospitals

County hospitals

County hospitals

**Stage 2**

Rural area

Urban area

Rural area

Urban area

Urban area

Rural area

Urban area

Rural area

All cities in Guangdong province were categorized into two levels: developed and developing cities;

Randomly selected two cities in each level

City 2: include 200 patients in total

City 2: include 200 patients in total

**Stage 1**

City 1: include 200 patients in total

City 1: include 200 patients in total

Developing cities: 2 cities

Developed cities: 2 cities
